# Prognostic Significance of CT-Attenuation of Tumor-Adjacent Breast Adipose Tissue in Breast Cancer Patients with Surgical Resection

**DOI:** 10.3390/cancers11081135

**Published:** 2019-08-08

**Authors:** Jeong Won Lee, Sung Yong Kim, Hyun Ju Lee, Sun Wook Han, Jong Eun Lee, Sang Mi Lee

**Affiliations:** 1 Department of Nuclear Medicine, International St. Mary’s Hospital, Catholic Kwandong University College of Medicine, 25 Simgok-ro 100 beon-gil, Seo-gu, Incheon 22711, Korea; 2 Department of Surgery, Soonchunhyang University Cheonan Hospital, 31 Suncheonhyang 6-gil, Dongnam-gu, Cheonan, Chungcheongnam-do 31151, Korea; 3 Department of Pathology, Soonchunhyang University Cheonan Hospital, 31 Suncheonhyang 6-gil, Dongnam-gu, Cheonan, Chungcheongnam-do 31151, Korea; 4 Department of Nuclear Medicine, Soonchunhyang University Cheonan Hospital, 31 Suncheonhyang 6-gil, Dongnam-gu, Cheonan, Chungcheongnam-do 31151, Korea

**Keywords:** breast cancer, Hounsfield unit, computed tomography, prognosis, adipose tissue

## Abstract

The purpose of this study was to evaluate the prognostic significance of computed tomography (CT)-attenuation of tumor-adjacent breast adipose tissue for predicting recurrence-free survival (RFS) in patients with breast cancer. We retrospectively enrolled 287 breast cancer patients who underwent pretreatment ^18^F-fluorodeoxyglucose (FDG) positron emission tomography (PET)/CT. From non-contrast-enhanced CT images of PET/CT, CT-attenuation values of tumor-adjacent breast adipose tissue (TAT HU) and contralateral breast adipose tissue (CAT HU) were measured. Difference (HU difference) and percent difference (HU difference %) in CT-attenuation values between TAT HU and CAT HU were calculated. The relationships of these breast adipose tissue parameters with tumor factors and RFS were assessed. TAT HU was significantly higher than CAT HU (*p* < 0.001). TAT HU, HU difference, and HU difference % showed significant correlations with T stage and estrogen receptor and progesterone receptor status (*p* < 0.05), whereas CAT HU had no significant relationships with tumor factors (*p* > 0.05). Patients with high TAT HU, HU difference, and HU difference % had significantly worse RFS than those with low values (*p* < 0.001). In multivariate analysis, TAT HU and HU difference % were significantly associated with RFS after adjusting for clinico-pathologic factors (*p* < 0.05). CT-attenuation of tumor-adjacent breast adipose tissue was significantly associated with RFS in patients with breast cancer. The findings seem to support the close contact between breast cancer cells and tumor-adjacent adipocytes observed with imaging studies.

## 1. Introduction

Adipose tissue is one of the major components of the human body, found all around the body [1]. In non-lactating human breast tissue, up to 56% of the total breast volume consists of adipose tissue [2]. Although adipose tissue is mainly composed of adipocytes, it is also comprised of various other kinds of cell types including immune cells, endothelial cells, pre-adipocytes, and fibroblasts [1,3]. For a long time, adipose tissue was considered to function as a simple storage organ of excessive energy; however, evidence showed that adipose tissue also function as an endocrine organ secreting hundreds of cell signaling proteins, called adipokines [4]. Previous studies published over the last decade have continuously demonstrated that adipose tissue could play a role in cancer growth, progression, and metastasis by secreting multiple kinds of adipokines, inducing inflammatory micro-environment, remodeling the extracellular matrix, and providing energy to tumor cells [1,3,5]. Furthermore, adipocytes in the close vicinity of cancer cells can have crosstalk with cancer cells, which increases the potential of tumor progression and metastasis [1,3,6]. Because of the close localization between mammary epithelium and breast adipose tissue, breast cancer is considered as one of the most representative malignancies that is in close contact with adipose tissue, along with gastric, colon, and ovary cancers [1,3]. In previous cell-culture and histopathological studies of breast cancer, it has already been proven that breast cancer cells have active interaction with peritumoral adipose tissue, which further enhances aggressiveness and invasiveness of breast cancer cells [1,3,7,8,9].

In recent clinical studies with imaging examinations, the clinical implication of adipose tissue features on diagnostic images has been investigated in various diseases [10,11,12]. In addition to the amount of adipose tissue, the qualitative characteristics of adipose tissue has also been studied using imaging modalities such as computed tomography (CT) and positron emission tomography (PET) [10,12,13,14,15]. One of the most commonly used imaging features representing qualitative changes in adipose tissue is CT-attenuation of adipose tissue measured on non-contrast-enhanced CT images [10,12,13,14]. In recent clinical studies with diverse kinds of malignancies, increased CT-attenuation on unenhanced CT images has shown a significant association with worse clinical outcomes, suggesting that increased CT-attenuation might reflect a microenvironment of adipose tissue which promotes cancer cell progression [12,13,14]. Considering the close localization between breast cancer and breast adipose tissue, the qualitative characteristics of peritumoral breast adipose tissue might have a significant association with the characteristics and clinical outcomes of breast cancer [1]. However, few studies have evaluated the clinical significance of the peritumoral adipose tissue environment using imaging studies [16].

In breast cancer, previous studies have shown the clinical significance of ^18^F-fluorodeoxyglucose (FDG) PET/CT for staging, differentiating molecular subtypes, and predicting clinical outcomes; therefore, currently, FDG PET/CT is widely used in patients with breast cancer [17,18,19,20]. In the present study, we used unenhanced CT images of pretreatment ^18^F-fluorodeoxyglucose (FDG) PET/CT for measuring CT-attenuation of tumor-adjacent breast adipose tissue and investigated whether it has a significant association with recurrence-free survival (RFS) in patients with breast cancer who underwent curative surgical resection.

## 2. Results

### 2.1. Patient Characteristics

Baseline characteristics of the 287 patients enrolled in the present study are shown in Table 1. Of the enrolled patients, 33 (11.5%) were histopathologically confirmed as having triple negative breast cancer. Neoadjuvant chemotherapy was performed in 32 patients (11.1%), and, after curative surgical resection, 282 patients (98.3%) received adjuvant treatments. The median interval between FDG PET/CT and initial treatment was 5.0 days (range, 2–14 days). On PET/CT image analysis of primary breast cancer, 89 patients (31.0%) had a metabolic tumor volume (MTV) of 0.0 cm^3^, because maximum standardized uptake values (SUVs) of the primary breast cancer were less than 2.50. The median follow-up duration of the patients was 55.1 months with a range of 6.1–88.9 months, and during the follow-up period, cancer recurrence was found in 30 patients (10.5%).

### 2.2. Breast Adipose Tissue Measurement

On the assessment of reproducibility of breast adipose tissue measurement, substantial agreement was shown between the two reviewers for CT-attenuation values, expressed in Hounsfield unit (HU), of tumor-adjacent breast adipose tissue (TAT HU) (concordance correlation coefficient: 0.953; 95% confidence interval: 0.934–0.968) and CT-attenuation values of contralateral breast adipose tissue (CAT HU) (concordance correlation coefficient: 0.951; 95% confidence interval: 0.924–0.972).

Among the enrolled patients, TAT HU was higher than CAT HU in 269 patients (93.7%), showing significant difference between TAT HU and CAT HU (*p* < 0.001). Body mass index (BMI) showed significant negative correlation with both TAT HU (r = −0.136, *p* = 0.001) and CAT HU (r = −0.274, *p* < 0.001) but showed weaker correlation with TAT HU and no significant correlation with the difference in CT-attenuation values between tumor-adjacent and contralateral breast adipose tissues (HU difference) (*p* = 0.231) and percent difference of CT-attenuation values between tumor-adjacent and contralateral breast adipose tissues (HU difference %) (*p* = 0.425).

**Figure 1:**
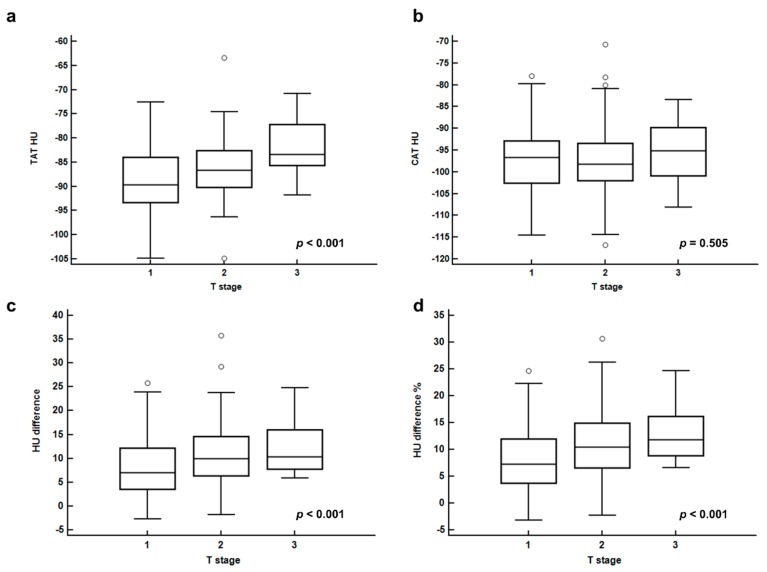
CT-attenuation of tumor-adjacent breast adipose tissue (TAT HU) (**a**), CT-attenuation of contralateral breast adipose tissue (CAT HU) (**b**), difference of CT-attenuation (HU difference) (**c**), and percent difference of CT-attenuation (HU difference %) (**d**), according to T stage.

In correlation analysis between breast adipose tissue parameters and tumor stage, increased CT-attenuation of peritumoral breast adipose tissue was related with advanced stage (Table 2). For T stage, significant differences of TAT HU, HU difference, and HU difference % were shown on Kruskal—Wallis test (*p* < 0.001 for all; Figure 1). On post-hoc analysis, each T stage group showed significant difference of TAT HU from each other, and HU difference and HU difference % in T2 and T3 stage groups were significantly higher than those in the T1 stage group (*p* < 0.05). For N stage, patients with regional lymph node metastasis had significantly higher HU difference and HU difference % than those with no lymph node metastasis (*p* < 0.001 for all). In contrast, there were no significant differences of CAT HU according to T stage and N stage (*p* > 0.05).

In correlation analysis with histopathological results, increased CT-attenuation of peritumoral breast adipose tissue was related with aggressive features of breast cancer (Table 2). Patients with negative estrogen receptor (ER) and progesterone receptor (PR) showed higher values of TAT HU, HU difference, and HU difference % than those with positive ER and PR, respectively (*p* < 0.05). Moreover, significant differences of HU difference and HU difference % were observed according to the histologic grade of the primary tumor, showing significantly higher HU difference and HU difference % in patients with histologic grade 3 tumors than in those with grade 1 and grade 2 tumors on post-hoc analysis (*p* < 0.05). In contrast, human epidermal growth factor receptor 2 (HER2) status and Ki67 expression status had no significant correlation with any breast adipose tissue parameters (*p* > 0.05).

In correlation analysis with FDG PET/CT parameters of primary breast cancer lesions, TAT HU, HU difference, and HU difference % showed significant but weak positive correlations with maximum SUV (r = 0.125, *p* = 0.034 for TAT HU, r = 0.266, *p* < 0.001 for HU difference, and r = 0.265, *p* < 0.001 for HU difference %) and MTV of primary tumor (r = 0.135, *p* = 0.023 for TAT HU, r = 0.248, *p* < 0.001 for HU difference, and r = 0.241, *p* < 0.001 for HU difference %).

**Figure 2:**
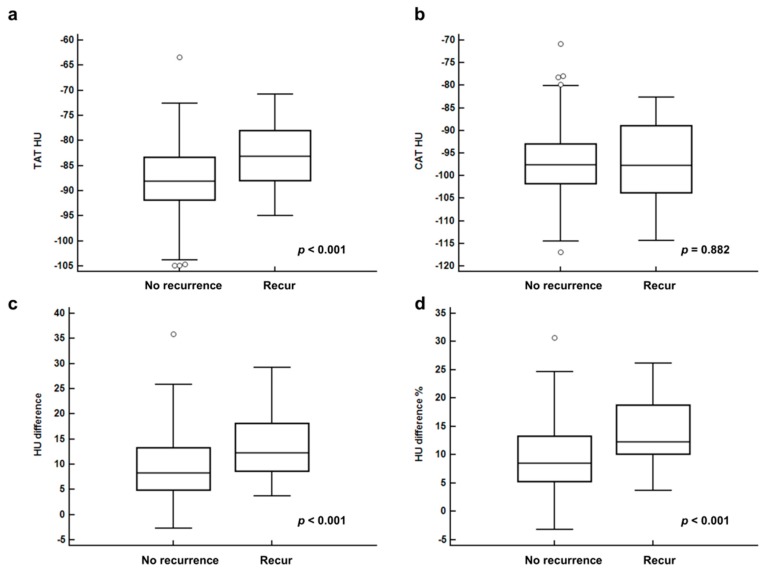
CT-attenuation of tumor-adjacent breast adipose tissue (TAT HU) (**a**), CT-attenuation of contralateral breast adipose tissue (CAT HU) (**b**), difference of CT-attenuation (HU difference) (**c**), and percent difference of CT-attenuation (HU difference %) (**d**) between patients with no recurrence and recurrence.

In comparison, the breast adipose tissue parameters of patients with recurrence showed significantly higher values of TAT HU, HU difference, and HU difference % than those with no recurrence (*p* < 0.001; Figure 2). In contrast, there is no significant difference in CAT HU between those two groups (*p* = 0.882; Figure 2)

### 2.3. Survival Analysis

The prognostic values of breast adipose tissue parameters for predicting RFS were evaluated using univariate Cox regression analysis along with clinico-histological factors and PET/CT parameters of the primary breast cancer. In univariate analysis, TAT HU, HU difference, and HU difference % were significantly associated with RFS (*p* < 0.001 for all; Table 3), while CAT HU showed no statistical significance (*p* = 0.786). Among clinico-histological factors and PET/CT parameters, T stage, N stage, histologic grade, ER status, PR status, Ki67 index, triple negative tumor, and maximum SUV and MTV of primary tumor showed significance in predicting RFS (*p* < 0.05; Table 3).

TAT HU, HU difference, and HU difference % were included in the multivariate analysis for RFS with the addition of eight different covariates in three different models (Table 4). TAT HU and HU difference % remained as significant positive predictors for RFS in all three models after adjustment for age, T stage, N stage, ER status, PR status, Ki67 index, maximum SUV, and MTV (*p* < 0.05). On the other hand, HU difference showed significant association with RFS in models with adjustment for age, T stage, N stage, ER status, PR status, and Ki67 index (*p* < 0.05) but failed to show statistical significance after correcting for maximum SUV and MTV (*p* = 0.054).

**Figure 3:**
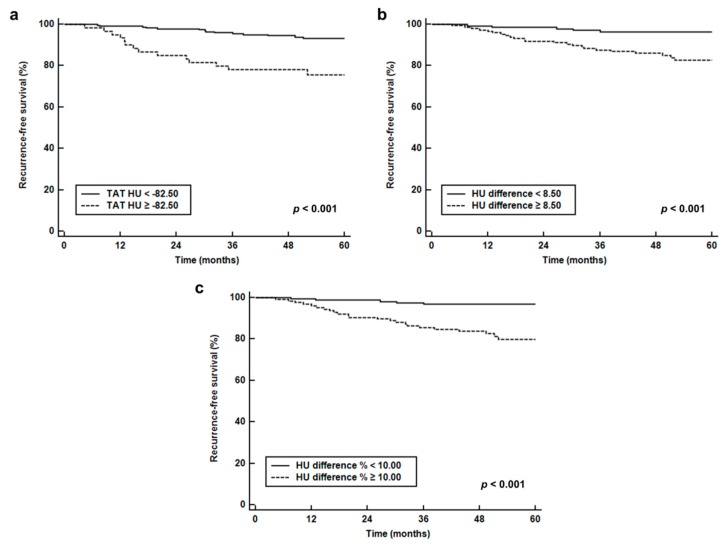
Survival curves. Recurrence-free survival stratified by CT-attenuation of tumor-adjacent breast adipose tissue (TAT HU) (**a**), difference of CT-attenuation (HU difference) (**b**), and percent difference of CT-attenuation (HU difference %) (**c**).

For Kaplan—Meier analysis, TAT HU, HU difference, and HU difference % were categorized into two groups according to the optimal cut-off values (−82.50 for TAT HU, 8.50 for HU difference, and 10.00 for HU difference %) determined by receiver operating characteristic (ROC) curve analysis. Kaplan—Meier analysis of enrolled patients stratified by TAT HU, HU difference, and HU difference % showed significantly worse RFS in patients with high values than in those with low values (*p* < 0.001 for all; Figure 3). Patients with high values of TAT HU (75.7% vs. 93.1%), HU difference (82.7% vs. 96.3%), and HU difference % (80.0% vs. 96.8%) showed lower 5-year RFS rates than those with low values.

While comparing recurrence rates based on the combination of T stage and TAT HU (Table 5), patients with high TAT HU showed a higher recurrence rate than those with low TAT HU, irrespective of T stage (5.3% vs. 0.9% for T1 stage; 34.1% vs. 12.7% for T2–T3 stage).

## 3. Discussion

In the present study, CT-attenuation of tumor-adjacent breast adipose tissue and difference of CT-attenuation between tumor-adjacent and contralateral breast adipose tissues showed significant association with T stage, tumor characteristics, and RFS in patients with breast cancer. In previous studies with histopathological analysis of resected breast cancer tissue, profound modifications of phenotype and biological features were found in the adipocytes that were present at the invasive front of the breast cancer cells [8,9]. The adipocytes in the vicinity of the breast cancer cells showed decreased cell size and less lipid content when compared to the adipocytes that are located far away from the breast cancer cells [8,9]. These alterations of adipocytes were reproduced in adipocytes which were co-cultivated with cancer cells, showing less differentiated features of adipocytes with decreased expression of adipocyte markers such as adiponectin and resistin and increased expression of matrix metalloproteinase 11, osteopontin, and pro-inflammatory cytokines including tumor necrosis factor-alpha and interleukin-6 [1,8,9,21]. These modified adipocytes are now called cancer-associated adipocytes [1,8,22]. The cancer-associated adipocytes are known to have an active bidirectional crosstalk with cancer cells, inducing fibrotic change in adipose tissue, stimulating tumor progression and metastasis by secreting multiple cytokines and adipokines, and releasing lipid content within the cell to provide source of energy to cancer cells [1,3,21,22,23]. Previous studies with breast cancer have also demonstrated that expressions of cancer-associated adipocytes-related protein are different according to the subtypes of breast cancer and the interactions between tumor cells and adipocytes can contribute to growth, proliferation, and epithelial-mesenchymal transition of breast cancer cells [23,24,25,26].

In a previous study that evaluated the correlation between CT-attenuation of adipose tissue and histopathological results of adipose tissue collected at necropsy in non-human primates, increased CT-attenuation of adipose tissue was significantly associated with smaller adipocytes and increased extracellular matrix fibrosis, which corresponded to the findings in the peritumoral adipose tissue affected by cancer cells [8,10,14]. Therefore, CT-attenuation of adipose tissue has been used as an imaging parameter for the qualitative alterations of adipose tissue in patients with malignant diseases [13,14]. In our study, we measured CT-attenuation in both tumor-adjacent and contralateral breast adipose tissue and tried to find out whether CT-attenuation of tumor-adjacent breast adipose tissue had different features compared to that of the contralateral side. The results of our study demonstrated that increased CT-attenuation was observed in tumor-adjacent breast adipose tissue compared to the contralateral side. Moreover, TAT HU, HU difference, and HU difference % had a significant correlation with the T stage, hormone receptor status, and FDG PET/CT parameters of the primary tumor, while CAT HU showed no significant association with them. These results suggested that qualitative changes had occurred in the peritumoral adipose tissue, which has a positive relationship with tumor aggressiveness.

Given that cancer-associated adipocytes conversely affect the growth and metastasis of cancer cells, it could be hypothesized that CT-attenuation of adipose tissue in patients with malignant diseases has significant prognostic significance for predicting clinical outcomes [5,7,13,14,15]. In previous studies, CT-attenuations of subcutaneous and visceral adipose tissue measured in the abdomen at the level of the lumbar spine were found to be significant predictors for disease progression-free survival and overall survival [12,13,14,15,27]. Previous studies consistently showed that increased CT-attenuation of adipose tissue was significantly associated with worse survival not only in malignant diseases that have close localization with adipose tissue such as pancreatic and prostate cancers, but also in malignant diseases that are remote from abdominal adipose tissue such as head and neck cancer and extremity sarcoma [12,13,14,27]. In a broad way, breast adipose tissue might be considered as a part of subcutaneous adipose tissue, but it permanently interacts with surrounding mammary epithelial cells consequently having distinctive characteristics from subcutaneous adipose tissue in other body compartments [7]. Considering that even the subcutaneous adipose tissue in remote location had association with cancer progression [13], it is no surprise that tumor-adjacent breast adipose tissue had significant association with cancer recurrence. The results of our study demonstrated that TAT HU and HU difference % were independently associated with RFS event after adjusting for age, tumor stage, ER and PR status, ki67 index, maximum SUV, and MTV, suggesting that perceiving the qualitative characteristics of peritumoral breast adipose tissue, as well as primary tumor features, might be of importance for predicting breast cancer recurrence after surgical resection. Furthermore, the combination of T stage and TAT HU can further improve the stratification of recurrence risk, suggesting that imaging features of tumor-adjacent breast adipose tissue might provide additional prognostic value to tumor stage. Recently, adipocytes have been considered as a new attractive treatment target for breast cancer [7,28,29]. The results of the present study might provide a clue for selecting candidates for future therapy that targets adipocytes in breast cancer patients.

Since the changes in the normal tissue surrounding breast cancer have drawn the attention of researchers, several recent studies have evaluated the clinical implication of imaging findings of tumor-adjacent breast parenchyma on magnetic resonance imaging (MRI) [30,31]. However, in the literature, only a single study has investigated the clinical significance of tumor-adjacent breast adipose tissue on imaging studies [16]. A previous study by Obeid et al. [16] retrospectively enrolled 63 patients with early breast cancer and investigated the association between imaging features of peritumoral breast adipose tissue on MRI images and axillary lymph nodal status. Similar to our study, they defined the peritumoral region as the area which is within a 1-cm spherical extension from the tumor contour. They generated peritumoral adipose volume using voxel intensity filtering and measured five imaging features of peritumoral tissue: mean voxel intensity, standard deviation of voxel intensity, total volume, fat-specific volume, and the fat ratio. Their study demonstrated that significant association was only shown between the fat ratio of peritumoral tissue and the ratio of positive-to-total axillary lymph nodes. On the other hand, the peritumoral fat ratio showed no significant correlation with other histopathologic features such as tumor grade, lymphovascular invasion, extracapsular extension, and tumor size, and the axillary lymph node status also showed no significant correlation with other imaging features of peritumoral tissue including mean voxel intensity. Because different imaging modalities were used in both studies, a direct comparison between our study and the previous studies would be limited. Although both CT and MRI are accurate and effective imaging modalities for adipose tissue measurement, each has different advantages and disadvantages [32,33]. CT is the most well-established method for adipose tissue measurement, and HU is consistent between images on difference scans, while CT scanning has the risk of exposure to high doses of ionizing radiation [32,33]. In contrast, adipose tissue measurements on MRI are expressed in arbitrary units that are difficult to compare between studies and are less reproducible than CT images [32,33]. Therefore, further studies using both CT and MRI in the same patients would be needed to compare the adipose tissue parameters of both modalities. Furthermore, CT-attenuation of only the breast adipose tissue was measured in our study, whereas voxel intensity of whole peritumoral breast tissue, including breast parenchyma and adipose tissue, was measured in the aforementioned study, which could cause differences in the results between the studies [16]. Nonetheless, our study also revealed no significant relationship between N stage and TAT HU, suggesting that the qualitative characteristics of tumor-adjacent breast tissue might have limited value for predicting axillary lymph node metastasis.

Recently, a dual-energy CT system has been introduced to improve tissue differentiation [34]. Using dual-energy CT, virtual non-contrast CT images can be synthesized, which could replace a precontrast CT scan, thereby reducing radiation exposure [34]. In previous studies, CT-attenuation values of various organs in virtual non-contrast CT images have shown close agreement with those in true unenhanced CT images [35,36]. Therefore, future studies are needed to clarify whether CT-attenuation of breast adipose tissue measured on virtual non-contrast CT images of dual-energy CT also has prognostic significance in breast cancer patients, which may form an imaging protocol for evaluating both tumor factors and tumor-adjacent breast tissue factors using a single CT scan.

There are several limitations to our study. Because the study was retrospectively performed in a single center and only the patients who had sufficient breast adipose tissue for the image analysis were included, selection bias is inevitable, which may limit the general application of the results of the present study. Furthermore, having a small number of patients who experienced recurrence could affect the results of survival analysis. Because of the retrospective nature of the study, we cannot compare the histopathological features of tumor-adjacent adipose tissue according to the CT-attenuation. Further studies with histopathological evaluation are needed to elucidate the mechanisms of our results. Lastly, as the present study is the first study to measure the tumor-adjacent breast adipose tissue, there is no established method of calculating CT-attenuation of breast adipose tissue [12,13]. A more sophisticated method, which could properly measure CT-attenuation of tumor-adjacent breast adipose tissue even in patients with a small amount of adipose tissue, would be helpful to confirm the association between breast adipose tissue parameters and clinical outcomes.

## 4. Materials and Methods

### 4.1. Study Population

This study was approved by the Institutional Review Board of Soonchunhyang University (Ethic code: SCHCA 2019-05-023, Date: 20 March 2019), and the requirement to obtain informed consent was waived by the board due to its retrospective nature. All procedures in this study were in accordance with the Declaration of Helsinki. Electronic medical records of 393 female patients who had biopsy proven invasive breast cancer and underwent pretreatment FDG PET/CT between February 2012 and December 2016 in our medical center were retrospectively reviewed. Among them, patients (1) who were diagnosed with distant metastasis on staging examinations, (2) who were diagnosed with ductal carcinoma in situ, (3) who had any kind of treatment before FDG PET/CT, (4) who had bilateral breast cancers, (5) who had a previous history of breast surgery, (6) who had a previous history of another malignancy, and (7) who were lost to follow-up within two years after the initial treatment without event were excluded from the study, and 355 patients met the initial eligibility criteria of the study. Of the 355 patients, 68 were excluded from the study during PET/CT image analysis due to insufficient breast adipose tissue for analysis (*n* = 55) and diffuse infiltration of breast cancer lesion (*n* = 13). Therefore, the remaining 287 female patients finally comprised the subjects of the study.

All enrolled patients underwent pretreatment staging examinations which consisted of blood tests, breast ultrasonography, breast MRI, bone scintigraphy, and FDG PET/CT. The BMI for each patient was calculated using the height and weight measured at the time of staging work-up. With the results of staging examinations, clinical TNM stage according to the 7th Edition of the American Joint Committee on Cancer staging system was determined for each patient. ER, PR, HER2, and Ki67 expression status were obtained from histopathological records. ER and PR positive tumors were defined as tumors with 10% or more positively stained cells using immunohistochemistry. A HER2 positive tumor was defined as a tumor with a 3+ score on immunohistochemistry or a tumor with gene amplification on fluorescence in situ hybridization. A tumor with 14% or more Ki67 expression using immunohistochemistry was defined as a Ki67 positive tumor. After staging examinations, all enrolled patients underwent curative surgical resection of breast cancer with or without neoadjuvant chemotherapy and/or adjuvant treatment. All patients were regularly followed up at intervals of 3–6 months with blood test and imaging studies. The duration of follow-up was calculated from the date of the initial treatment to the last date of clinical follow-up at our medical center or to the occurrence of cancer recurrence.

### 4.2. Image Analysis

All patients underwent FDG PET/CT scanning from the skull base to the proximal thigh in a supine position using a Biograph mCT 128 scanner (Siemens Healthcare, Knoxville, TN, USA) after at least six hours fasting. One hour after intravenous injection of approximately 4.07 MBq/kg of FDG, a non-contrast-enhanced attenuation correction CT scan was performed at 100 mA and 120 kV_p_ with a slice thickness of 5 mm followed by a PET scan at 1.5 min per bed position. PET images were reconstructed using point-spread-function modeling and time-of-flight reconstruction (2 iterations and 21 subsets) with attenuation correction.

**Figure 4:**
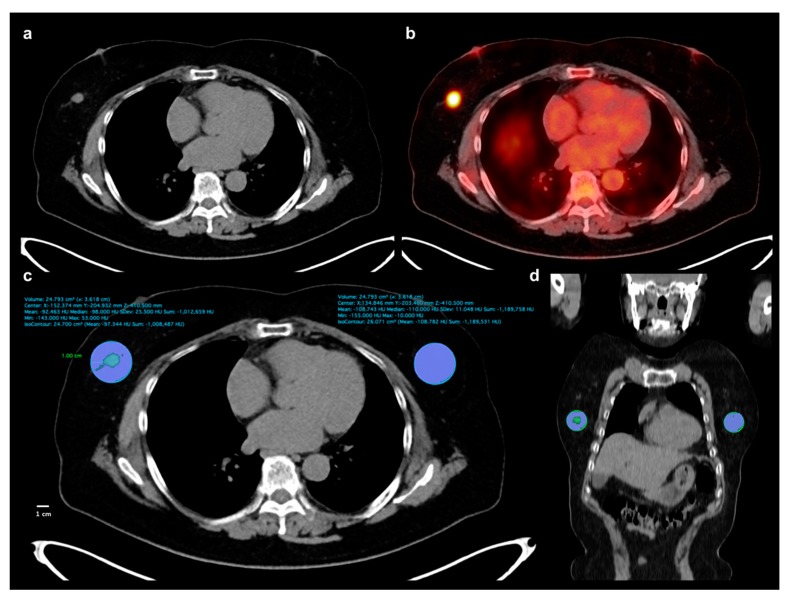
Measurement example of CT-attenuation of tumor-adjacent breast adipose tissue and contralateral breast adipose tissue. A 62-year-old woman underwent FDG PET/CT for staging work-up of right breast cancer, histopathologically confirmed as invasive ductal carcinoma. A mass lesion with intensely increased FDG uptake is observed in outer aspect of right breast, (**a**,**b**), showing maximum SUV of 8.44. A spheroid-shaped volume-of-interest that includes peritumoral breast tissue within a 1-cm distance from the tumor margin was manually drawn over the ipsilateral breast tissue, (**c**,**d**). Another spheroid-shaped volume-of-interest of the same size was drawn over the contralateral breast tissue in the same quadrant of the breast, (c,d). Within the volume-of-interests drawn in bilateral breast tissues, the area of breast adipose tissue defined as an area with CT-attenuation range between −200 and −50 HU was automatically delineated. Mean CT-attenuation value of breast adipose tissue was measured for each VOI (c): −92.463 HU for tumor-adjacent breast adipose tissue and −108.743 for contralateral breast adipose tissue.

Two expert readers independently reviewed FDG PET/CT images of all patients without knowing clinico-pathological and follow-up results. A United States Food and Drug Administration-approved medical image viewer (OsiriX MD 10.0.3, Pixmeo, Geneva, Switzerland) was used for image analyses. For breast adipose tissue measurement, non-contrast-enhanced CT images of FDG PET/CT were used. A total of four parameters (TAT HU, CAT HU, HU difference, and HU difference %) were measured from breast adipose tissue. We used a 1-cm distance from the tumor margin for defining tumor-adjacent breast adipose tissue. A spheroid-shaped volume-of-interest (VOI) that includes the entire breast cancer lesion and surrounding breast tissue within a 1-cm distance to the tumor margin was manually drawn (Figure 4). Afterwards, another spheroid-shaped VOI of the same size was drawn over the contralateral breast tissue in the same quadrant of the breast tissue (Figure 4). A CT-attenuation range of −200 and −50 HU was used to define breast adipose tissue within VOIs. The mean CT-attenuation value of the area within the CT-attenuation range of breast adipose tissue was measured for each VOI and defined as TAT HU and CAT HU. Using TAT HU and CAT HU, HU difference and HU difference % were calculated as follows: (HU difference) = (TAT HU) − (CAT HU) while (HU difference %) = ((CAT HU) − (TAT HU))/(CAT HU) × 100. Using FDG PET images, we measured two metabolic parameters of primary breast cancer lesion, maximum SUV and MTV. A spheroid-shaped VOI was drawn over the primary cancer lesion on fused PET/CT images including the whole tumor lesion. The maximum SUV of the primary breast cancer lesion was measured. A total volume of voxels that had SUV of 2.50 or greater within VOI was measured and defined as MTV of primary cancer lesion.

### 4.3. Statistical Analysis

To assess the reproducibility of measurement of TAT HU and CAT HU between the two readers, concordance correlation coefficients for TAT HU and CAT HU were calculated. Paired *t* test was performed to assess the difference between TAT HU and CAT HU. A Student’s *t* test and Kruskal—Wallis test were performed to compare the differences in four breast adipose tissue parameters between groups. After performing the normality test, Spearman rank correlation coefficients were calculated for the breast adipose tissue parameters with regard to BMI and maximum SUV and MTV of the primary breast cancer lesion. The primary endpoint of the present study was RFS. Univariate and multivariate survival analyses using the Cox proportional hazards regression model were performed to assess the association between variables and RFS. Of the four breast adipose tissue parameters, those with a *p*-value of less than 0.10 in univariate analysis were included in multivariate analysis. In the multivariate analysis of breast adipose tissue parameters, three different models with eight different covariates (age, T stage, N stage, ER status, PR status, Ki67 index, maximum SUV, and MTV) were constructed. The significances of the association between breast adipose tissue parameters and RFS were evaluated after adjusting for these eight covariates. For breast adipose tissue parameters that showed statistical significance in univariate survival analysis, the optimal cut-off values were determined using ROC curve analysis, and survival curves for RFS according to the cut-off values were estimated using the Kaplan—Meier method. All statistical analyses were performed using MedCalc Statistical Software version 19.0.3 (MedCalc Software bvba, Ostend, Belgium). A *p*-value of <0.05 was considered statistically significant.

## 5. Conclusions

TAT HU and HU difference % were independent predictors for RFS in patients with breast cancer, suggesting that qualitative alterations of tumor-adjacent breast adipose tissue may have significant association with tumor recurrence. TAT HU and HU difference % had a significant relationship with T stage and hormone receptor status of breast cancer, and patients with increased CT-attenuation of peritumoral adipose tissue had worse survival. Although further studies are warranted to confirm the results, the results of our study seem to support the close interaction between breast cancer cells and tumor-adjacent adipocytes observed with imaging studies.

## Figures and Tables

**Table 1:** Baseline characteristics of patients (*n* = 287).

Characteristics		Number (%)	Median (Range)
Age (years)			52 (30–85)
Body mass index			23.8 (16.4–35.2)
Menopausal status	Premenopausal	108 (37.6%)	
	Postmenopausal	179 (62.4%)	
Histopathology	Intraductal carcinoma	252 (87.8%)	
	Intralobular carcinoma	35 (12.2%)	
T stage	T1	136 (47.4%)	
	T2	128 (44.6%)	
	T3	23 (8.0%)	
N stage	N0	190 (66.2%)	
	N1	58 (20.2%)	
	N2	21 (7.3%)	
	N3	18 (6.3%)	
Tumor size (cm)			2.0 (0.4–8.0)
Histologic grade	Grade 1	67 (23.4%)	
	Grade 2	143 (49.7%)	
	Grade 3	77 (26.9%)	
Estrogen receptor statue	Positive	213 (74.2%)	
	Negative	74 (25.8%)	
Progesterone receptor status	Positive	177 (61.7%)	
	Negative	110 (38.3%)	
HER2 status	Positive	142 (49.5%)	
	Negative	145 (50.5%)	
Ki67 expression status	Positive	177 (61.7%)	
	Negative	110 (38.3%)	
Maximum SUV of primary tumor			4.06 (1.10–35.59)
MTV of primary tumor (cm^3^)			1.14 (0.0–235.30)
TAT HU			−87.69 (−104.88–−63.36)
CAT HU			−97.54 (−116.85–−70.74)
HU difference			8.66 (−2.67–35.84)
HU difference %			8.94 (−3.15–30.67)
Neoadjuvant chemotherapy	Yes	32 (11.1%)	
	No	255 (88.9%)	
Adjuvant treatment	CTx + RTx + HTx	139 (48.4%)	
	RTx + HTx	82 (28.6%)	
	CTx + HTx	17 (5.9%)	
	CTx + RTx	3 (1.0%)	
	HTx	24 (8.4%)	
	CTx	16 (5.6%)	
	RTx	1 (0.3%)	
	No	5 (1.7%)	

HER2, human epidermal growth factor receptor 2; SUV, standardized uptake value; MTV, metabolic tumor volume; TAT HU, CT-attenuation of tumor-adjacent breast adipose tissue; CAT HU, CT-attenuation of contralateral breast adipose tissue; HU difference, difference of CT-attenuation; HU difference %, percent difference of CT-attenuation; CTx, chemotherapy; RTx, radiotherapy; HTx, hormonal therapy.

**Table 2:** Relationship between breast cancer adipose tissue parameters and clinico-histological factors.

Clinico-Histological Factors		TAT HU	CAT HU	HU Difference	HU Difference %
T stage	T1	−88.95 ± 6.53	−96.95 ± 6.61	7.99 ± 6.06	8.08 ± 6.02
	T2	−86.33 ± 5.72	−97.15 ± 7.22	10.82 ± 6.69	10.87 ± 6.23
	T3	−82.67 ± 5.68	−95.43 ± 7.39	12.76 ± 6.18	13.14 ± 5.66
	*p*-value	<0.001 *	0.505 *	<0.001 *	<0.001 *
N stage	N0	−87.64 ± 6.26	−96.95 ± 6.82	8.63 ± 6.43	8.76 ± 6.27
	N1-3	−86.56 ± 6.56	−98.06 ± 7.97	11.61 ± 6.34	11.63 ± 5.92
	*p*-value	0.176	0.203	<0.001	<0.001
Histologic grade	Grade 1	−87.62 ± 7.21	−96.28 ± 7.61	8.65 ± 7.11	8.76 ± 6.75
	Grade 2	−87.65 ± 6.23	−96.87 ± 6.71	9.23 ± 6.42	9.32 ± 6.21
	Grade 3	−86.27 ± 5.85	−97.58 ± 6.84	11.21 ± 6.09	11.29 ± 5.83
	*p*-value	0.288 *	0.391 *	0.013 *	0.013 *
ER status	Positive	−88.03 ± 6.26	−96.76 ± 6.91	8.73 ± 6.34	8.81 ± 6.09
	Negative	−85.11 ± 6.25	−97.36 ± 7.07	12.25 ± 6.46	12.36 ± 6.15
	*p*-value	<0.001	0.528	<0.001	<0.001
PR status	Positive	−87.94 ± 6.47	−96.60 ± 6.76	8.16 ± 5.73	8.34 ± 5.69
	Negative	−86.22 ± 6.09	−97.23 ± 7.06	12.01 ± 7.08	11.97 ± 6.58
	*p*-value	0.026	0.318	<0.001	<0.001
HER2 status	Positive	−87.54 ± 5.71	−97.56 ± 6.34	10.02 ± 6.79	10.03 ± 6.49
	Negative	−87.02 ± 6.97	−96.29 ± 7.45	9.27 ± 6.29	9.43 ± 6.10
	*p*-value	0.488	0.122	0.336	0.426
Ki67 expression status	Positive	−86.72 ± 6.10	−96.88 ± 6.83	10.16 ± 6.76	10.25 ± 6.42
	Negative	−88.01 ± 7.01	−96.72 ± 7.37	8.72 ± 6.25	8.82 ± 6.17
	*p*-value	0.115	0.856	0.083	0.076
Recurrence	No recur	−87.74 ± 6.27	−96.90 ± 6.74	9.16 ± 6.37	9.25 ± 6.17
	Recur	−83.35 ± 6.00	−97.10 ± 8.62	13.75 ± 6.71	13.85 ± 5.91
	*p*-value	<0.001	0.882	<0.001	<0.001

* Performed using Kruskal—Wallis test.; ER, estrogen receptor; PR, progesterone receptor; HER2, human epidermal growth factor receptor 2; TAT HU, CT-attenuation of tumor-adjacent breast adipose tissue; CAT HU, CT-attenuation of contralateral breast adipose tissue; HU difference, difference of CT-attenuation; HU difference %, percent difference of CT-attenuation.

**Table 3:** Univariate analysis for recurrence-free survival.

Variables		*p*-Value	Hazard Ratio (95% CI)
Age (1-year increase)		0.562	1.01 (0.98–1.04)
BMI (1 kg/m^2^ increase)		0.283	0.93 (0.82–1.06)
Menopausal status (pre vs. post)		0.799	0.91 (0.44–1.89)
T stage	T1 stage	-	1.00
	T2 stage	0.001	11.24 (2.62–48.25)
	T3 stage	<0.001	37.90 (8.17–175.80)
N stage (N0 vs. N1-3)		0.004	2.92 (1.42–6.09)
Histologic grade	Grade 1	-	1.00
	Grade 2	0.873	1.10 (0.34–3.58)
	Grade 3	0.008	4.42 (1.48–13.16)
ER status (positive vs. negative)		<0.001	3.41 (1.66–7.00)
PR status (positive vs. negative)		<0.001	5.22 (2.32–11.74)
HER2 status (positive vs. negative)		0.254	0.65 (0.31–1.36)
Ki67 index (negative vs. positive)		0.005	7.79 (1.85–32.83)
Triple negative tumor (no vs. yes)		0.011	3.00 (1.28–7.03)
Maximum SUV (1.0 increase)		<0.001	1.08 (1.03–1.13)
MTV (1.0 cm^3^ increase)		<0.001	1.03 (1.02–1.04)
TAT HU (1.0 HU increase)		<0.001	1.10 (1.05–1.16)
CAT HU (1.0 HU increase)		0.786	0.99 (0.94–1.05)
HU difference (1.0 HU increase)		<0.001	1.10 (1.05–1.16)
HU difference % (1.0% increase)		<0.001	1.12 (1.06–1.18)

BMI, body mass index; ER, estrogen receptor; PR, progesterone receptor; HER2, human epidermal growth factor receptor 2; SUV, standardized uptake value; MTV, metabolic tumor volume; TAT HU, CT-attenuation of tumor-adjacent breast adipose tissue; CAT HU, CT-attenuation of contralateral breast adipose tissue; HU difference, difference of CT-attenuation; HU difference %, percent difference of CT-attenuation; HU, Hounsfield units; CI, confidence interval.

**Table 4:** Multivariate models for recurrence-free survival.

Models		TAT HU	HU Difference	HU Difference %
		(1.0 HU Increase)	(1.0 HU Increase)	(1.0% Increase)
Model 1 *	*p*-value	0.012	0.001	0.001
	Hazard ratio	1.08	1.07	1.09
	(95% CI)	(1.02–1.15)	(1.02–1.19)	(1.02–1.15)
Model 2 †	*p*-value	0.018	0.044	0.033
	Hazard ratio	1.08	1.06	1.07
	(95% CI)	(1.01–1.16)	(1.00–1.12)	(1.01–1.14)
Model 3 ‡	*p*-value	0.014	0.054	0.038
	Hazard ratio	1.09	1.05	1.07
	(95% CI)	(1.02–1.16)	(1.00–1.11)	(1.00–1.13)

* Adjusted for age, T stage and N stage; † Adjusted for age, T stage, N stage, estrogen receptor status, progesterone receptor status, and Ki67 index; ‡ Adjusted for age, T stage, N stage, estrogen receptor status, progesterone receptor status, Ki67 index, maximum standardized uptake value, and metabolic tumor volume; CI, confidence interval; TAT HU, CT-attenuation of tumor-adjacent breast adipose tissue; HU difference, difference of CT-attenuation; HU difference %, percent difference of CT-attenuation; HU, Hounsfield units.

**Table 5:** Recurrence rates according to the combination of T stage and TAT HU.

TAT HU		T Stage	
		T1 Stage	T2–T3 Stage
TAT HU	<−82.50 HU	1/117	14/110
		(0.9%)	(12.7%)
	≥−82.50 HU	1/19	14/41
		(5.3%)	(34.1%)
	*p*-value	0.260	0.003

TAT HU, CT-attenuation of tumor-adjacent breast adipose tissue; HU, Hounsfield units.
